# Neuronal activity in the hub of extrasynaptic Schwann cell-axon interactions

**DOI:** 10.3389/fncel.2013.00228

**Published:** 2013-11-25

**Authors:** Chrysanthi Samara, Olivier Poirot, Enric Domènech-Estévez, Roman Chrast

**Affiliations:** Department of Medical Genetics, University of LausanneLausanne, Switzerland

**Keywords:** peripheral nervous system, Schwann cell, axon-glia interaction, neuronal activity, microarray, neuronal support

## Abstract

The integrity and function of neurons depend on their continuous interactions with glial cells. In the peripheral nervous system glial functions are exerted by Schwann cells (SCs). SCs sense synaptic and extrasynaptic manifestations of action potential propagation and adapt their physiology to support neuronal activity. We review here existing literature data on extrasynaptic bidirectional axon-SC communication, focusing particularly on neuronal activity implications. To shed light on underlying mechanisms, we conduct a thorough analysis of microarray data from SC-rich mouse sciatic nerve at different developmental stages and in neuropathic models. We identify molecules that are potentially involved in SC detection of neuronal activity signals inducing subsequent glial responses. We further suggest that alterations in the activity-dependent axon-SC crosstalk impact on peripheral neuropathies. Together with previously reported data, these observations open new perspectives for deciphering glial mechanisms of neuronal function support.

## Introduction

Neurons generate and propagate action potentials (APs) over long distances along their axons. Their functional and structural integrity depend on their partnership with adjacent glial cells. Glia confers trophic and metabolic support, regulates neuronal structure, insulates axons, controls the neuronal environment and has immunoprotective role. In the peripheral nervous system (PNS) the majority of these functions are exerted by Schwann cells (SCs) (Griffin and Thompson, 2008; Nave, 2010). Most SCs are aligned along peripheral axons of the sensory, motor, and autonomic nervous system, and are either myelinating (mSCs) or non-myelinating. The latter include immature SCs (iSCs) and mature non-myelinating SCs (nmSCs) in Remak bundles. Furthermore, the PNS contains perineuronal satellite cells enwrapping the neuronal soma, perisynaptic SCs in neuromuscular junctions (NMJs), and SCs of sensory transducers.

SCs were assumed to be passive in nature. However, experimental observations have radically challenged this concept. Converging evidence suggests that SCs are excitable, able to sense neuronal activity and generate appropriate feedback responses to support and control neuronal function. This dynamic reciprocal activity-dependent SC-neuron communication is the focus of our perspective. Although the majority of respective information has stemmed from studies on NMJs (Feng and Ko, 2007), we review here only the less well-studied extrasynaptic interactions between SCs and active axons under physiological and pathological conditions. We put into perspective the current literature with some of our recent data, and point to future directions in the field.

## Detection of axonal activity by SCs

Intercellular interactions can be mediated through electrical fields generated in a cell and depolarizing neighboring cells bearing voltage sensors (ephaptic communication), via paracrine signaling, and by physical coupling, for instance through adhesion molecules or gap junctions (GJs). Indications exist for the utilization of all three means in activity-dependent interactions among PNS neurons and glia.

### Signals transmitted by active axons

**Figure 1:**
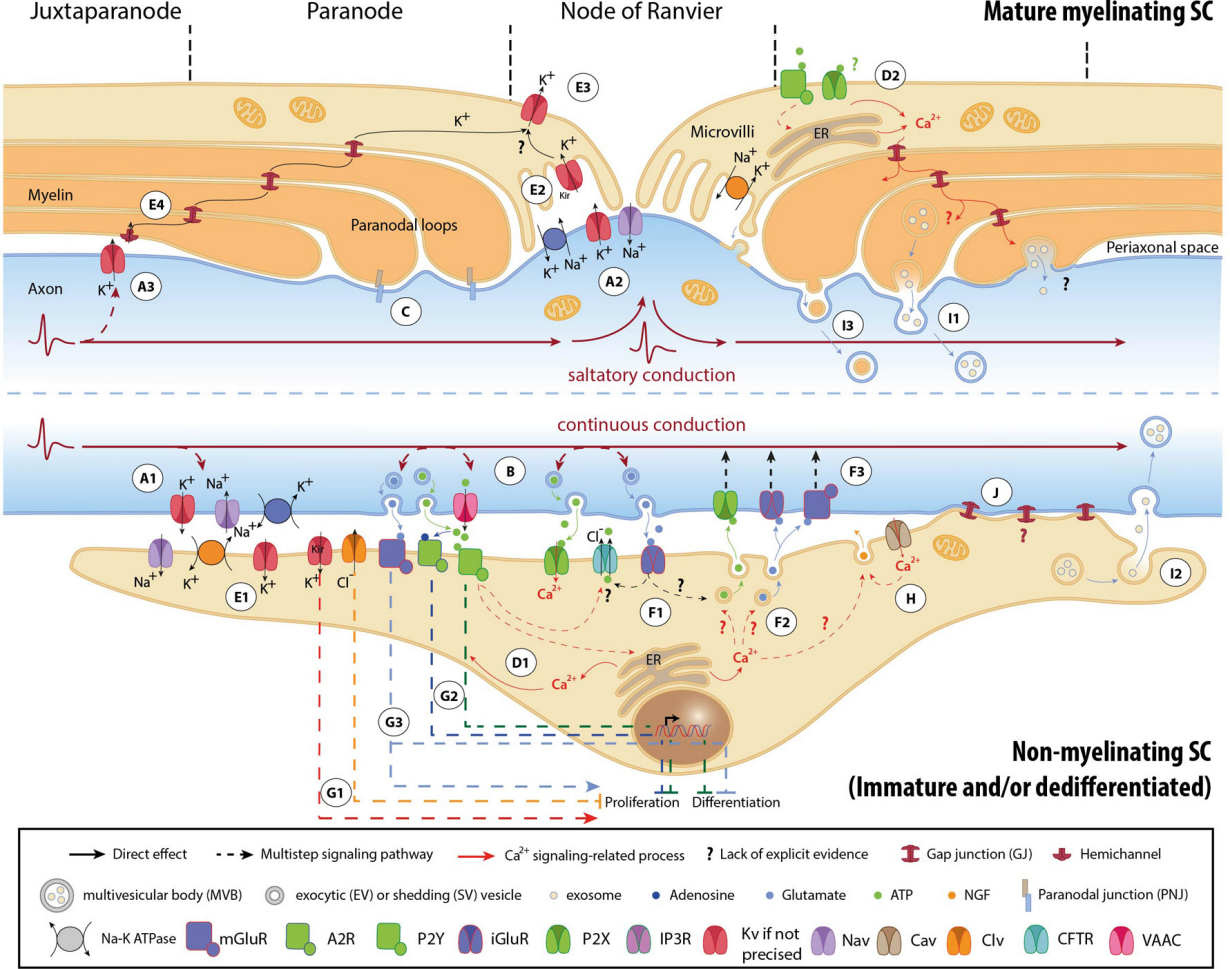
**Mechanisms involved in activity-dependent axon-Schwann cell bilateral communication**. Schematic representation of the different molecules and mechanisms described in myelinated (upper part) and non-myelinated (lower part) PNS fibers. **(A)** Ephaptic communication through ion flows across the plasmalemma of unmyelinated **(A1)** and myelinated axons **(A2, A3)**. **(B)** Paracrine signaling from axons to SCs. **(C)** Physical coupling between axons and mSCs. **(D)** SC Ca^2+^ transients developing after neuronal stimulation. In nmSCs activation of purinergic receptors leads to increase of cytoplasmic Ca^2+^ due to influx from the extracellular space, or efflux from intracellular stores **(D1)** (Stevens et al., 1998; Stevens and Fields, 2000; Stevens et al., 2004). mSCs express both P2X and P2Y receptors, and also respond to ATP stimulation by Ca^2+^ increase **(D2)** (Mayer et al., 1998; Grafe et al., 1999). Indications suggest that Ca^2+^ transients expand in the whole paranodal region through GJs (Toews et al., 2007). The origin of ATP in mature myelinated fibers, however, is not clear. High ATP levels, sufficient to activate glial receptors, are probably generated only during high frequency activity or after injury. **(E)** K^+^ buffering and ion homeostasis. K^+^ uptake by nmSCs through the Na^+^/K^+^ pump and K_*V*_ channels **(E1)** (Robert and Jirounek, 1994). In mSCs, inward rectifying K_*V*_ channels (IRK1/Kir2.1 and IRK3/Kir2.3), and Na^+^/K^+^ ATPases are concentrated in microvilli **(E2)**, where massive increase of K^+^ occurs during neuronal activity (Mi et al., 1996; Baker, 2002). Abaxonal K_*V*_1.5 channels in the nodal area may further assist to K^+^ removal **(E3)** (Mi et al., 1995; Baker, 2002). In juxtaparanodal and internodal regions, axonal K_*V*_1 channels may act in conjunction with closely apposed SC hemichannels and with GJs of the Schmidt-Lanterman incisures (SLIs) for the same purpose (**E4**, see also **A3**) (Altevogt et al., 2002; Mierzwa et al., 2010; Nualart-Marti et al., 2013). **(F)** Paracrine signaling from SCs to axons. Activation of P2Y and AMPA receptors acts in a positive feedback loop, triggering ATP release by nmSCs, through vesicular exocytosis or via ion transporters, such as CFTR **(F1)** (Liu and Bennett, 2003; Liu et al., 2005). Administration of ATP on proliferating SCs induces secretion of the excitatory amino acids Glu and aspartate, via intracellular Ca^2+^ store-dependent mechanisms **(F2)** (Jeftinija and Jeftinija, 1998). ATP and excitatory amino acids can reciprocally bind to ionotropic and metabotropic Glu-, and P2X-receptors on unmyelinated peripheral axons and influence their excitability **(F3)** (Agrawal and Evans, 1986; Kinkelin et al., 2000; Carlton et al., 2001; Irnich et al., 2001). **(G)** Regulation of SC fate by neuronal activity through activation of ion channels **(G1)** (Wilson and Chiu, 1993; Pappas and Ritchie, 1998; Sobko et al., 1998), purinergic metabotropic P2Y_1_ receptors and A2_*A*_ GPCRs by ATP and its metabolite adenosine **(G2)** (Stevens and Fields, 2000; Stevens et al., 2004; Fields and Burnstock, 2006), and of mGluRs **(G3)** (Saitoh and Araki, 2010). **(H)** Neurotrophic axonal support by SCs. **(I)** Vesicular transfer of molecules from SCs to axons. Exosomes, which are enclosed in multivesicular bodies (MVB), move from mSCs to axons through cytoplasmic-rich regions like the SLIs and paranodal domains **(I1)**, or can be released from dedifferentiated/iSCs close to neuronal growth cones after injury **(I2)** (Lopez-Verrilli and Court, 2012). Shedding vesicles (SVs) are directly generated from SC plasma membrane evaginations usually in microvilli and paranodal areas of mSCs, and can fuse or be endocytosed by axons **(I3)** (Court et al., 2008; Cocucci et al., 2009; Lopez-Verrilli and Court, 2012). **(J)** Potential direct transfer route of SC molecules via GJs. Abbreviations: Ca_*V*_, voltage-gated Ca^2+^ channel; Cl_*V*_, voltage-gated Cl^−^ channel; K_*V*_, voltage-gated K^+^ channel; Kir, inwardly rectifying K^+^ channel, Na_*V*_, voltage-gated Na^+^ channel; CFTR, Cystic Fibrosis Transmembrane conductance Regulator; VAAC, Volume-Activated Anion Channel; A2R, adenosine receptor 2; P2X and P2Y, purinergic receptor; iGluR, ionotropic glutamate receptor; mGluR, metabotropic glutamate receptor; GPCR, G-protein coupled receptor; NGF, nerve growth factor; ER, Endoplasmic Reticulum.

APs are generated by activation of specific voltage–gated Na^+^ (Na_*V*_) and K^+^ (K_*V*_) channels, and propagate autoregeneratively along axons. In non-myelinated fibers APs travel successively through ion channels expressed all along the axons (Figure 1A1) (Debanne et al., 2011). In myelinated fibers, ion channels are mainly clustered in nodal (Na_*V*_1.6, K_*V*_7.2-3) and juxtaparanodal (JPN, K_*V*_1.1-2) regions, and conduction is saltatory (Figures 1A2,A3) (Debanne et al., 2011; Buttermore et al., 2013). Ion flows generate local currents in the periaxonal space, which can influence surrounding cells via ephaptic coupling (Debanne et al., 2011).

Firing axons also release neurotransmitters (Figure 1B). Electrical or chemical stimulation *in vitro* induces extrasynaptic axonal ATP secretion through volume-activated anion channels (VAACs), via vesicular pathways (Verderio et al., 2006; Fields and Ni, 2010). Electrical stimulation (ES) evokes vesicular release of glutamate (Glu) along DRG axons, at least in cocultures with oligodendrocytes (Wake et al., 2011). Observations demonstrating exocytosis of large dense core vesicles by chemically depolarized axons of trigeminal ganglion neurons further support the concept of activity-induced extrasynaptic axonal secretion (Sobota et al., 2010).

In addition, axons are physically coupled to SCs via adhesive junctions, such as the paranodal junctions (PNJs) (Figure 1C) (Buttermore et al., 2013). The expression of specific axonal adhesion molecules is under regulation by ES in a pattern-specific manner (Itoh et al., 1997).

### Detection of axonal signals by SC activity sensors

SC responses to neuronal activity were initially recorded on the squid giant axon by electrophysiology (Evans et al., 1991). ES of axons or perfusion of neurotransmitters induced SC membrane hyperpolarization (Evans et al., 1991). Similar responses have been also reported in vertebrates, mainly in the form of SC Ca^2+^ transients that develop subsequently to ES of myelinated and unmyelinated fibers (Figures 1D1,D2) (Brunet and Jirounek, 1994; Lev-Ram and Ellisman, 1995; Mayer et al., 1999).

mSCs and nmSCs express molecules, which allow them to respond to electrical or chemical axonal stimuli (Figure 1). SC “activity sensors,” including voltage- and ligand-gated ion channels, transporters, pumps, G-protein coupled receptors (GPCRs), connexins (Cx) of hemichannels and GJs, have been detected at mRNA and protein levels *in vivo* (animal tissues or human biopsies), *ex vivo* (nerve preparations) and/or *in vitro* (SC cultures), using biochemical and functional approaches (Dememes et al., 1995; Dezawa et al., 1998; Mayer et al., 1998; Verkhratsky and Steinhauser, 2000; Altevogt et al., 2002; Baker, 2002; Fields and Burnstock, 2006; Loreti et al., 2006; Magnaghi et al., 2006; Saitoh and Araki, 2010; Procacci et al., 2012; Nualart-Marti et al., 2013). A summary of the so far-identified SC receptors and ion channels is presented in Table 1.

**Table 1:** **Expression and regulation of potential SC activity sensors**.

**Families**		**Subtypes**	**Expression in SCs**		**Transcriptional regulation ^p^**			
					**During development**		**In neuropathy models**	
			**Previously published data^a^^−^^o^**	**Microarray data^p^**	**Up**	**Down**	**Up**	**Down**
Potassium channels^*a*^^,^^*b*^	Voltage-gated	Delayed rectifier, A-type, outward-rectifying, inward-rectifying, slowly activating	Kv1.1, Kv1.2,Kv1.5, Kv1.4, Kv2.1, Kv3.1b, Kv3.2,Kv7.3, Kv7.5 in iSCs or SN, inactivating A-type and delayed-rectifier current in soma	Kv1.1, Kv1.2, Kv1.6, Kv2.1, Kvβ 1, Kvβ 2, Kvβ 3, minK-like, Kv5.1 Kv7.5, Kv11.1, Kv11.3, Kv6.2	Kv1.1, Kv1.2, Kvβ 1, Kv5.1, Kv7.5	Kv2.1, Kvβ 3, Kv3.4, minK-like Kv7.5	Kv1.6	–
	Inwardly rectifying	IRK, Kir2.x, subfamily J	Kir2.1/IRK1, Kir2.3,/IRK3, in nmSC soma and mSC microvilli	Kir2.2/IRK2	Kir2.2/IRK2	–	–	Kir2.2/IRK2
		Kir4.x	–	Kir4.1	–	–	–	–
		Kir6.x, KATP, ATP-sensitive	–	Kir6.1/UKATP-1	Kir6.1	Kir6.1	–	–
	Calcium activated	BK channel	Maxi-K^+^ current in iSC soma	KCa1.1, KCa4.1	KCa4.1	KCa1.1	–	–
		SK channel	–	KCa2.2, KCa2.3, KCa3.1	KCa3.1	KCa3.1	–	KCa3.1
	Tandem pore domain	TWIK, TREK, TASK, TALK, THIK, TRESK	–	Twik-1, Trek-1, Task-2, Twik-2, Thick1	Twick-1, Thick-1, Twick-2, Task-1	Trek-1, Task-2, Task-1	–	Twick-1, Trek-1
Voltage-gated sodium channels ^*a*^^,^^*b*^		TTX-sensitive	Nav1.2,3,7 in iSCs, current in iSC soma	Navβ 1, Navβ 2, Navβ 3^*^, Navβ 4	Navβ 1	Navβ 3^*^	Navβ 1	–
		TTX-resistant	Current in iSC soma	–	–	–	–	–
		NaG	NavX in iSCs and nmSCs	NavX^**^	NavX^**^	–	–	NavX^**^
Voltage-gated calcium channels^*a*^^,^^*b*^	Alpha subunits	T-type VGCCs	Current in iSC soma	Cav3.1 (low), Cav3.2	–	Cav3.2	Cav3.2	–
		L-type	Current in iSC soma	Cav1.1	–	–	–	–
		P/Q	–	Cav2.1	–	–	–	–
	Auxiliary subunits	Auxiliary subunits	–	γ1, β 1, β 3, β 4, α2δ 1	β 3	γ1	–	–
Chloride channels^*a*^^,^^*b*^		Voltage-gated	Current in iSC soma	Clcn2–4, and 7	Clcn2, Clcn3	–	–	Clcn2
		Large-conductance (VDAC1)	Current in iSC soma and myelin vesicles	VDAC1^**^	–	VDAC1^**^	–	–
TRP channels		TRPC, TRPV, TRPM	–	Trpm3, Trpm5	–	Trpm3, Trpm5	–	–
Purinergic receptorsa ^*a*^^−^^*e*^		P2X^*b*^^,^^*c*^^,^^*p*^	P2X1-4, P2X7 in iSC soma and in paranodal region mSCs	P2X1, 4, 5, 7	P2X5, P2X7	–	–	–
		P2Y^*c*^^,^^*p*^	P2Y1, P2Y2, P2Y12, P2Y13 in iSCs, current in mSC paranodes	P2Y1, 2, 6, 13, and 14	P2Y2	P2Y13	P2Y13, and 14	–
		P1^*c*^	A2a, A2b in iSCs, current in iSC soma	A1	A1	–	–	–
Glutamate receptors	Ionotropic^*f*^^−^^*h*^	AMPA receptors	GluA2-4 in vestibular mSCs, current in iSC soma, SN and iSCs	GluA1, GluA2, GluA3	GluA3	GluA2	GluA1, GluA3	–
		Kainate receptors	iSC soma	GluK2, GluK3	GluK3	–	GluK2	–
		NMDA receptors	iSC soma	GluN1	GluN1	–	–	–
		Delta receptors	–	GluD2	–	–	–	GluD2
	Metabotropic^*i*^	mGluR	mGluR in iSC soma	–	–	–	–	–
Adrenergic receptors^*f*^		A1 and A2	–	Adrα2a, Adrβ 2	Adrβ 2	–	–	Adrα2a
		Nicotinic	–	α1,and 9, β 1, γ	γ	–	–	–
		Muscarinic^*h*^	M1-4 in iSCs, current in iSC soma	M3	–	–	–	–
GABA receptors^*k*^^,^^*l*^		GabaA^*i*^^,^^*j*^	α1-3, β 1-3, γ2 in SN, and SCs, current in iSC soma	GabaAβ 3	–	GabaAβ 3	GabaAβ 3	–
		GabaB^*j*^	GabaB1, and 2 in nmSCs, and iSCs, current in iSC soma	GabaB1	–	–	–	–
GAP-junctions^*l*^^−^^*n*^		Cx^*k*^^−^^*m*^	Cx29,32, and 43 in mSCs; Cx32, and in iSCs, Cx 29 in iSCs	Cx29,30,32,37,40,43,45, and 47	Cx29,32, and 47	Cx37,40, and 45	Cx30	Cx43

Previously published data (based on biochemical and functional studies) regarding expression of potential SC activity sensors are summarized in the middle-left column called “Previously published data.” Data generated through analysis of SN microarray experiments (Verdier et al., 2012) are presented in the middle-right column called “Microarray data.” Right part of the table demonstrates transcriptional regulation of depicted sensors during development and in peripheral neuropathy, based on analyses of data initially presented in (Verdier et al., 2012) (Up: upregulated transcripts, Down: downregulated transcripts). Detailed description of data processing and the complete list of significantly modulated genes can be found in the original paper (Verdier et al., 2012) and in its supporting information (http://onlinelibrary.wiley.com/doi/10.1002/glia.22305/suppinfo). The complete data set is accessible through the ArrayExpress database (accession number: E-MTAB-944; http://www.ebi.ac.uk/arrayexpress/). Asterisks (^*^) denote transcripts, which have been previously described in adult intact (^*^) or injured (^**^) DRG axons Willis et al., 2007; Gumy et al., 2011, and may thus be detected (at least partially) due to contamination by axonal mRNA.

a Verkhratsky and Steinhauser, 2000;

b Baker, 2002;

c Fields and Burnstock, 2006;

d Verderio et al., 2006;

e Colomar and Amedee, 2001;

f Liu and Bennett, 2003;

g Fink et al., 1999;

h Dememes et al., 1995;

i Saitoh and Araki, 2010;

j Loreti et al., 2006;

+k Magnaghi et al., 2006;

l Procacci et al., 2012;

m Dezawa et al., 1998;

n Altevogt et al., 2002;

o Nualart-Marti et al., 2013;

p *Verdier et al., 2012*.

### Developmental regulation of SC activity sensors

Responsiveness of SCs to neuronal activity is developmentally regulated. Downregulation of K_*V*_ channel expression during early myelination, and clustering to microvilli in mature mSCs is a characteristic example (Figure 1) (Wilson and Chiu, 1990). However, scarce evidence exists regarding the developmental regulation of other SC activity sensors. To gain further insight, we analyzed microarray data previously published by our group (Verdier et al., 2012), on wild type (WT) mouse sciatic nerve (SN) at different developmental stages. Since the analyzed samples are highly enriched in SCs, we expect that the majority of the detected sensors represent SC molecules and do not derive from axon specific transcripts (Willis et al., 2007; Gumy et al., 2011), (see also Table 1). Our results -summarized in Table 1- corroborate and complete existing data, confirming the expression of specific voltage- (e.g., Na_*V*_, K_*V*_, voltage-gated Ca^2+^ channels; Ca_*V*_, Cl_*V*_), and ligand-gated (e.g., purinergic P2X and ionotropic glutamate receptors -iGluRs) ion channels, and of GPCRs (e.g., purinergic P2Y, muscarinic acetylcholine receptors, GABA_B_ receptors) (Fink et al., 1999; Baker, 2002; Loreti et al., 2006; Magnaghi et al., 2006). In addition, they reveal previously non-described mammalian SC expression of nicotinic acetylcholine receptors and TRP channels. Apart from the known regulation of K^+^ channels, our data suggest that expression of Na^+^, Ca^2+^, Cl^−^, and TRP channels, purinergic receptors and iGluRs is also significantly regulated during development.

These transcriptional modulations could result as adaptations of SCs to different neuronal firing modes. The reduction and restriction of K_*V*_ channels in mSC microvilli most likely corresponds to the need for K^+^ buffering mainly in nodal regions (see also paragraph “K^+^ uptake by SCs”) (Wilson and Chiu, 1990; Baker, 2002). In addition, nmSC inwardly rectifying K^+^ (Kir)-currents and T-type Ca_*V*_ depend on axonal firing (Konishi, 1994; Beaudu-Lange et al., 2000). Given that the firing patterns of nerve fibers change during maturation (Fitzgerald, 1987), we speculate that developmental regulation of SC activity sensors could be a direct glial response to axonal activity alterations. Alternatively, it may reflect mere phenotypic changes during SC maturation.

Further SC responses to neuronal activity will be the focus of the following paragraphs.

## SC responses to axonal activity signals

Detection of axonal activity by glial sensors enables SCs to develop appropriate responses and -in a feedback loop- regulate the function of underlying axons. We will discuss the nature and the potential biological significance of those SC responses, focusing particularly on their direct (via ion balance regulation, neurotransmitter secretion and myelination) or indirect (by conferring metabolic support) impact on axonal activity.

### Regulation of axonal excitability

#### K^+^ uptake by SCs

During prolonged neuronal activity, Na^+^ and K^+^ ions tend to accumulate in the axoplasm and in the periaxonal space respectively. Maintenance of neuronal excitability requires maintenance of ion homeostasis and fast restoration of the axonal resting potential. Both nmSC and mSCs contribute to it by buffering extracellular K^+^ ions, mainly through the activity of Na^+^/K^+^ pumps and K_*V*_ channels (for more details see Figure 1E).

#### SC neurotransmitter secretion

Axonal firing leads to ATP and Glu release in the periaxonal space (Figure 1B, see also paragraph Signals transmitted by active axons) (Verderio et al., 2006; Fields and Ni, 2010; Wake et al., 2011). By activating P2Y and AMPA receptors on iSCs and nmSCs, these neurotransmitters reciprocally trigger secretion of ATP and the excitatory amino acids Glu and aspartate from SCs, via ion channels or vesicular mechanisms (Figures 1F1,F2) (Jeftinija and Jeftinija, 1998; Liu and Bennett, 2003; Liu et al., 2005). SCs may also secrete the inhibitory neurotransmitter GABA, known to modulate peripheral fiber excitability, but whether its secretion is induced by neuronal activity has not been determined (Morris et al., 1983; Carr et al., 2010; Magnaghi et al., 2010). SC-released neurotransmitters exert local effects on axonal excitability (Carlton et al., 2001; Irnich et al., 2001) (Figure 1F3). Moreover, they may initiate signals that propagate electrically or via retrograde axonal transport toward neuronal cell bodies, affecting soma signaling processes and gene expression (Itoh et al., 1997; Amir and Devor, 2003; Chen et al., 2012).

#### SC differentiation and myelination

Myelin production by SCs leads to the organization of enwrapped axons into distinct structural domains with highly specialized patterns of ion channel expression (Salzer, 2003; Buttermore et al., 2013). Internodes, electrically insulated by myelin layers with low electrical capacitance, alternate with ion-rich nodes of Ranvier, where APs are generated, so that fast and energy efficient saltatory stimulus propagation is achieved (Figures 1A–C). Hence, neuronal activity effects on SC differentiation can have significant consequences on axon excitability and AP conduction.

Early during development, firing of unmyelinated PNS fibers induces ionic imbalances and neurotransmitter secretion, which affect iSC maturation and myelin production. Cl_*V*_ and still unidentified K^+^ channels regulate iSC mitosis by modulating the SC membrane potential (Wilson and Chiu, 1993; Pappas and Ritchie, 1998; Sobko et al., 1998) (Figure 1G1). *In vitro* ES of embryonic DRG neurons, at low frequencies that mimic DRG spontaneous spiking at early developmental stages, leads to activation of purinergic signaling pathways and subsequent inhibition of both SC proliferation and differentiation (Figure 1G2) (Stevens and Fields, 2000; Stevens et al., 2004). Myelination reduction by low-frequency ES has been further attributed to downregulation of the axonal adhesion molecule L1 (Stevens et al., 1998). Glu and GABA also modulate SC maturation (Figure 1G3) (Magnaghi et al., 2006; Saitoh and Araki, 2010; Procacci et al., 2012). However, although GABA is known to be released by SCs (see paragraph “Neurotransmitter secretion”), its extrasynaptic secretion from PNS axons has not been demonstrated.

Few existing experimental data suggest that neuronal activity controls myelination also in the mature PNS. Subfunctional soleus nerve fibers in hindlimb-unloaded rats exhibit reduced myelin thickness (Canu et al., 2009). Administration of ATP modulates myelin lipid constitution in frog SN preparations (Kutuzov NP et al., 2013). Whether and how neuronal function is affected by these changes requires further investigation.

### Trophic and metabolic support of neurons

Neuronal activity depends on the maintenance of axonal integrity and energetic status. Both nmSCs and mSCs provide neurotropic and metabolic support to adjacent neurons (Griffin and Thompson, 2008; Nave, 2010). This support is under the control of axonal activity. In response to ES and ATP, cultured SCs secrete nerve growth factor (NGF) and brain-derived neurotropic factor (BDNF), respectively, promoting axonal growth (Figure 1H) (Verderio et al., 2006; Huang et al., 2010). In addition, chemical depolarization triggers vesicular transport of molecules from SCs to axons (Figure 1I) at least in invertebrates (Eyman et al., 2007). Reported molecular cargo of SC-to-axon transported vesicles includes ribosome-bound mRNA, cytoskeletal components and heat-shock proteins (Court et al., 2008; Cocucci et al., 2009; Lopez-Verrilli and Court, 2012). Their exact contributions to axonal function under physiological conditions are still unknown.

Although information regarding glia-to-axon metabolic support in the PNS is scarce, inferences could be made from CNS data. Neuronal activity triggers exosome transfer of metabolic enzymes from oligodendrocytes to neurons (Fruhbeis et al., 2013), as well as release of lactate from astrocytes and uptake by neurons (Barros, 2013). Similar energy transfer processes may occur in the PNS. ES in SN increases O_2_ uptake and glucose consumption, and SCs seem to be the main metabolic SN niche (Heller and Hesse, 1961). Moreover, *in vivo* genetic disruption of mitochondria energy production in otherwise functional mouse SCs severely impairs the structure and function of peripheral fibers (Viader et al., 2011; Funfschilling et al., 2012), suggesting that there may be SC-to-neuron energy transfer also in the PNS. However, its characterization, and potential regulation by neuronal activity await further investigation.

## Pathogenic disruption of activity-dependent SC–axon communication

Significant insight into the physiological significance of the SC-axon cross-talk and its contribution to the maintenance of axonal excitability and function has been obtained by studies on PNS pathologies, such as inflammatory (e.g., chronic inflammatory demyelinating polyneuropathies), metabolic (e.g., diabetes) or genetic (e.g., Charcot-Marie Tooth, -CMT) diseases, and injury.

### Dysregulation of SC activity sensors in pathologies

Peripheral neuropathies have been linked to dysregulation of SC activity sensors. Overexpression of P2X7 receptors may have a causative role in CMT1A patient demyelination due to Ca^2+^ overload (Nobbio et al., 2009). Moreover, P2X7 activation induces BDNF secretion and activates K^+^ and Cl^−^ conductances, through Big K^+^ channels and more likely via the cystic fibrosis transmembrane conductance regulator CFTR (Colomar and Amedee, 2001; Verderio et al., 2006). Interestingly, Cl^−^ imbalance leads to axonal loss with primary or secondary dysmyelination in patients and animal models with dysfunctional CFTR or the K^+^-Cl^−^ cotransporter KCC3 (Sun et al., 2010; Reznikov et al., 2013). Certain CMTX patients carry mutations in Cx32, which may lead to increased currents through the Cx32-hemichannel and to subsequent nerve damage (Abrams et al., 2002; Nualart-Marti et al., 2013). Dysregulation of SC sensors (e.g., upregulation of K_*V*_ and Na_*V*_ channels) also occurs after injury (Chiu, 1988).

To further investigate the contribution of SC activity sensor regulation to PNS dysfunctions, we checked for respective transcriptional modulations in our previously published microarray data on SN endoneuria from three mouse models of peripheral neuropathy: the *Scap* and *Lpin1* conditional knockouts (KOs), which have defective lipid biosynthesis and exhibit PNS hypomyelination and progressive demyelination, respectively, and the *Pmp22* total KO, which lacks the myelin protein PMP22 and is a model of Hereditary Neuropathy with Liability to Pressure Palsy (Table 1) (Adlkofer et al., 1995; Nadra et al., 2008; Verheijen et al., 2009; Verdier et al., 2012). With the exception of TRP channels and acetylcholine receptors, we are able to detect expression changes in all families of SC sensors. Their potential role in pathogenesis can be inferred from existing data. Upregulation of K^+^ channels may interfere with SC ability to buffer K^+^ ions or be associated with increased proliferation of dedifferentiated SCs (Wilson and Chiu, 1990, 1993) (Figures 1E2,G1). Upregulation of T-type Ca_*V*_3.2 channels could trigger NGF release, in order to support underlying affected axons (Figure 1H) (Huang et al., 2010). A time-course analysis of the transcriptionally regulated genes during the progress of pathology, in conjunction with functional studies, would be necessary to delineate their potential destructive or protective roles in the development of neuropathy.

### Disruption of neuronal activity due to myelin defects

Myelin defects are a common feature of various peripheral neuropathies. Studies on animal models of demyelinating diseases (e.g., CMT1A, CMT1B, CMT1C, and CMTX) have demonstrated that myelin impairments affect neural influx conduction and axonal excitability through different mechanisms, including decreased electrical isolation of the axolemma, the exposure, redistribution or abnormal expression of voltage-gated ion channels, and the potential change from saltatory to continuous conduction (Brismar, 1981b, 1982; Rasminsky, 1982; Meiri et al., 1986; England et al., 1990, 1996; Schwarz et al., 1991; Rasband et al., 1998; Neuberg et al., 1999; Devaux and Scherer, 2005; Moldovan et al., 2011; Lee et al., 2013). Aberrant expression of nodal Na_*V*_ channels and nodal or juxtaparanodal K_*V*_ channels, has been confirmed in patients with CMT1A and CMT4C (Nodera et al., 2004; Arnaud et al., 2009). Computational simulations in combination with experimental observations correlate those demyelination-induced changes with alterations in axonal excitability and impulse propagation, leading to negative or positive clinical symptoms. Alteration in axonal domains can induce decreased excitability and even conduction failure underlying negative symptoms of peripheral neuropathies, such as muscle weakness (Brismar, 1981a,b; Cappelen-Smith et al., 2001; Nodera et al., 2004; Jani-Acsadi et al., 2008; Coggan et al., 2010; Moldovan et al., 2011). Alternatively, demyelination can lead to axonal hyperexcitability, spontaneous ectopic spiking and cross excitation of neighboring axons (by ephaptic coupling or crossed afterdischarge), leading to positive symptoms like neuropathic pain (Calvin et al., 1982; Rasminsky, 1982; Lisney and Pover, 1983; Lisney and Devor, 1987; Gillespie et al., 2000; Wallace et al., 2003; Gemignani et al., 2004; Coggan et al., 2010).

### SC support of dysfunctional axons

Axonal dysfunctions in pathologies and animal models with impaired SCs may also occur secondary to or without myelin abnormalities (Gabreels-Festen et al., 1992; Griffiths et al., 1998; Chen et al., 2003; Nave, 2010), indicating the implication of myelin-unrelated mechanisms. Failure of trophic or metabolic glia-to-neuron support may be one such mechanism. Glial support is particularly critical for neuropathic fibers, which have increased metabolic requirements, due to their decreased propagation efficiencies (Shrager and Rubinstein, 1990; De Waegh et al., 1992; Kirkpatrick and Brady, 1994; Moldovan et al., 2011). Glycogen stored in mSCs is utilized to provide neurons with lactate particularly during aglycemia (Brown et al., 2012). Likewise, exosome transport of metabolic enzymes from oligodendrocytes to axons is required to sustain neuronal survival and function under stress conditions (Fruhbeis et al., 2013), while vesicular transfer of ribosomes from mSCs is prominent in injured fibers, and promotes regeneration (Court et al., 2008, 2011; Lopez-Verrilli et al., 2013). Mutations affecting exosome-mediated intercellular communication have been recently described in CMT1C patients (Zhu et al., 2013). Direct transfer of SC molecules via GJs has been suggested in regenerating nerves (Figure 1J) (Dezawa et al., 1998). Apparently, under pathological conditions, SCs need to adjust their physiology in order to maintain the integrity and function of suffering axons.

To investigate whether glia-to-axon support mechanisms are affected in our *Scap*, *Lpin1*, and *Pmp22* mouse models, we checked for transcriptional regulation of genes involved in cellular metabolism (excluding lipid metabolism, since its dysregulation is expected in the *Scap* and *Lpin1* KOs) and vesicle trafficking, and for genes encoding potential SC exosome or other vesicular cargo (Lopez-Verrilli and Court, 2012; Fruhbeis et al., 2013). Results, depicted in Table S1, reveal changes in genes of all categories. Detailed analyses at both glial and neuronal levels are required to check the potential positive or negative impact of those alterations on the diseased phenotype, especially since some of the depicted transcripts are also present in axons (Willis et al., 2007; Gumy et al., 2011).

## Conclusions and perspectives

Neuronal activity plays a central role in the extrasynaptic communication between peripheral axons and SCs. SCs express proteins that allow them to detect signals produced by firing axons. Our microarray data indicate that the list of SC activity sensors may be more extensive than currently known, thus providing indications for novel axonal activity signals. Detection of those signals permits SCs to adjust their physiology, so as to sufficiently support and control neuronal activity. Although this reciprocal interaction is constantly required to sustain the PNS function, it becomes particularly critical in transitional periods, during development or under pathology-induced stress. By identifying SC activity sensor- and neuronal support-genes that are regulated during development and/or PNS disease, we attempt to shed light on mechanisms mobilized by SCs to cover the altered needs and increased requirements of the challenged nervous system. More questions, however, arise, especially regarding the potential contribution of neuronal activity signals to these regulations, their nature, the downstream signaling pathways mediating SC responses, and the role of the latter in the maintenance of neuronal integrity and the regulation of axonal function. Characterization of respective mechanisms can be facilitated by implementation of recently developed microfluidic compartmentalized cell culture technologies that enable cell-specific analyses and application of advanced microscopy techniques (Taylor et al., 2005). Combination with *in vitro* ES via conventional electrodes or microelectrode array platforms could be used to investigate the neuronal activity dependence and relevance of SC molecules and signaling pathways (Kanagasabapathi et al., 2011; Yang et al., 2012; Jokinen et al., 2013; Malone et al., 2013). Apart from revealing new modulators of myelination, we expect that such studies will also contribute to the understanding of myelin-independent mechanisms of SC-to-neuron crosstalk.

## Author contributions

Chrysanthi Samara and Olivier Poirot, concept and design, data analysis, and interpretation, manuscript writing; Enric Domènech-Estévez, manuscript writing; Roman Chrast, concept, and design, final approval of manuscript, financial support.

### Conflict of interest statement

The authors declare that the research was conducted in the absence of any commercial or financial relationships that could be construed as a potential conflict of interest.

## Supplementary material

Table S1 **Transcriptional regulation of genes encoding potential SC-to-neuron support molecules in mouse models of peripheral neuropathies**. Re-analyzed microarray data were originally generated by characterization of endoneurial samples from adult, 56 days-old *Scap*, *Lpin1*, and *Pmp22* knockout mice. The grouping in the categories of “Metabolism” and “Vesicle trafficking” was based on Gene Ontology, whereas grouping in the “Exosome-exocytic vesicle cargo” category was performed by manual annotation based on (Lopez-Verrilli and Court, 2012; Fruhbeis et al., 2013). For more information regarding the experiments and data analysis, see legend of Table 1 and (Verdier et al., 2012). Asterisk (^*^) indicates transcripts that have been previously described in axons of DRG neurons (Willis et al., 2007; Gumy et al., 2011).
