# Supplementary material for: Neuronal activity in the hub of extrasynaptic Schwann cell-axon interactions
Source: Front Cell Neurosci. 2013 Nov 25;7:228. doi: 10.3389/fncel.2013.00228 (PMC3839048; doi:10.3389/fncel.2013.00228)
Supplement: Table S1 — Transcriptional regulation of genes encoding potential SC-to-neuron support molecules in mouse models of peripheral neuropathies. Re-analyzed microarray data were originally generated by characterization of endoneurial samples from adult, 56 days-old Scap, Lpin1, and Pmp22 knockout mice. The grouping in the categories of “Metabolism” and “Vesicle trafficking” was based on Gene Ontology, whereas grouping in the “Exosome-exocytic vesicle cargo” category was performed by manual annotation based on (Lopez-Verrilli and Court, 2012; Fruhbeis et al., 2013). For more information regarding the experiments and data analysis, see legend of Table 1 and (Verdier et al., 2012). Asterisk (*) indicates transcripts that have been previously described in axons of DRG neurons (Willis et al., 2007; Gumy et al., 2011). [file DataSheet1.PDF]

SC molecules potentially implicated in regulation/ support of neuronal function		Transcriptional regulation in mouse models of peripheral neuropathies	
**Category**	**Sub-category**	**Upregulated**	**Downregulated**
Metabolism	Glycolysis/ Gluconeogenesis	Atf3, Atf4, Hk2	Acss2, Aldh2, Alsoa, Aldoc, Hk1, Pfkm
	Pentose phosphate pathway	Pgd, Pgls	Tkt
	Polysaccharide metabolism	Hyal1, Hyal2, Ppp1ca	Chi3l1
	Oligosaccharide metabolism	Akr1b8, Ddx54, Gcs1, Hexa, Man2b1, Pfkfb4	Pmm1, Pygb, Sord
	Proteoglycan metabolism	Glce, Gns, Hs2st1	Chst1, Chst2, Extl1
	One-carbon compound metabolism	Car10, Mthfd1l, Mthfd2	
	Oxidative phosphorylation	Atp5c1, Atp6v1b2, Cox5a, Ndufa1, Ndufa4, Tcirg1	Atp6v0e2, Cox7a1, Ndufa5, Ndufc1
	Oxidation reduction	Loxl1, Prdx5, Qsox1, Vat1	AI427515, BC026585, Bdh1, Cbr2, Cyzl1, Cyp2d22, Cyp4f14, Cyp4v3, Foxred2, Pcyox1l
	Carbohydrate transport	Slc2a3, Slc4a7, Slc35b1-2-3, Slc35c1	Slc2a1, Slc2a6, Slc35e3
	Other	Crym, Nat11, Uap1l1	231001406Rik, Acly, AI790298, Enpp4, Fggy, Fn3k, Gpd1, Lctl, Nat8l, Pdk4, Prei4, Stbd1
Vesicle trafficking	Vesicle-mediated transport	Bet1l, Colt1b, Copa, Copb2, Copg, Lman1, Sec13, Sec14c, Stx11, Vps26a, Vps37b	Arf3, Bcap29, Mapk8ip1, Rab2, Stx7, Vamp8, Vapb
	Endocytosis	Rab34, Sh3gl3	Hip1r, Mrc1, Pacsin3, Rabgef1, Syp, Ston1, Trip10
	Exocytosis	Myo5a, Stxbp2, Syt4	Cplx1, Scamp1, Snap25, Stxbp1, Unc13c
Exosome-exocytic vesicle cargo	Enzymes (oxidative stress)	Gstp1, Prdx2	
	Enzymes (metabolism)		Aldoa, Gpd1, Sirt2, Tkt
	Chaperones	Hsp90b1	Hsp90ab1
	Ribosomal proteins	Rpl13a, Rps27l, Exosc4, Mrpl33, 20, 17, 12 & 34, Rrp12, Rrbp1	Mrps6
	Other glia-identified cargo		Prnp, Flot1, Vegfb, Npepl1
